# Bibliometric Analysis on Bibliometric Studies of Case Reports in the Medical Field

**DOI:** 10.7759/cureus.29905

**Published:** 2022-10-04

**Authors:** Sonia M Gupta, Waqar M Naqvi, Kalyani N Mutkure, Ashish Varma, Sumit Thakur, Roshan Umate

**Affiliations:** 1 Research, NKP Salve Institute of Medical Sciences and Research Centre, Nagpur, IND; 2 Rehabilitation, Perfection Physiotherapy Clinic, Nagpur, IND; 3 Department of Paediatrics, Jawaharlal Nehru Medical College, Datta Meghe Institute of Medical Sciences, Wardha, IND; 4 Department of Paediatrics, David Ferguson Neonatal Unit, Newport, GBR; 5 Research and Development, Jawaharlal Nehru Medical College, Datta Meghe Institute of Medical Sciences, Wardha, IND

**Keywords:** citations, scientometric, medical sciences, scopus, pubmed, case reports, bibliometrics

## Abstract

The studies on bibliometric analyses of case reports usually give valuable information regarding various aspects of case reports but lack investigation analysing these publications. This is the first-ever study to examine the bibliometric articles on case reports; hence, it is hypothesized to provide a valuable contribution to this gap. PubMed and SCOPUS databases were searched, and a total of 119 articles were obtained, but only five were analyzed matching the inclusion criteria. The keywords involved in the search were “Bibliometrics”, “analysis”, “case reports”, "case series”, and “articles” whereas, the time range in which the case reports were searched for was between 2011-2021. Common parameters from these five articles were employed for bibliometric analysis, which included publication year, publication type, the number of case reports per article, theme or subject of the article, citation, and impact factor (IF). Out of the five articles identified, four were published in 2021. One out of five was a case report, and the rest were review-type of articles. The overall citation number of these articles was less than 10, and the IF of these articles was between 0-0.007. The number of citations of the articles was in a period of one to two years or less than one year. A comprehensive overview of the parametrises, as well as the recent trends that are being used to conduct bibliometric analysis on case reports was acquired.

## Introduction and background

Bibliometrics is closely associated with scientometrics as well as informatics, which deals with quantitative and statistical analyses of the literature, that is, collective writings of a given subject area [1]. Such analyses are necessary to evaluate scientific and clinical activity in a particular field that provides essential qualitative as well as quantitative information on their current status in the progression of that particular branch. Important scientific observations that are sometimes undetectable in clinical trials can be detected using the information provided by case reports. This insightful information helps broaden our knowledge and leads to better patient care [2]. Hence, such evidence-based practices are important because they make certain pieces of clinical knowledge, such as rare diseases, unusual clinical symptoms, side effects of different treatments, and therapeutic effects, accessible. 

Bibliometrics of case reports or case series that are published aid in the assessment of their impact on clinical practice, which may provide knowledge of similarities and differences in symptoms of a particular disease in terms of investigating tools and therapeutic interventions. It identifies the ongoing research problems and can also help in determining the impact factor (IF) and citations of the case reports, which is a major metric for determining the value of the article. Periodical evaluation and bibliometric analyses of case reports provide awareness of topics that are popular and frequently studied. 

Important contributions have been made by many researchers who have published their bibliometric analyses of case reports, but there is no such publication yet to analyze these studies together [3-7]. Bibliometric analysis of such bibliometric studies on case reports is the first ever study in the medical field and is thought to provide a valuable contribution to this research gap. Hence, we conducted and reported the first bibliometric analysis of bibliometrics on case reports.

## Review

Data extraction and search approach

Articles on bibliometric analysis of case reports were identified and retrieved from Elsevier's SCOPUS and PubMed databases. The data extraction was conducted in two stages. First, the published bibliometric articles were identified, while the second stage involved screening the articles and excluding those that were not purely case-oriented.

Since bibliometric articles may not essentially contain the term “bibliometric” in the title or abstract, a primarily advanced search was carried out in the PubMed database using ‘AND’ for the English language, in addition to ‘NOT’ for publication type, in order to obtain more thorough search results. Simple keywords such as “Bibliometrics”, “analysis”, “case reports”, "case series”, and “articles” were used, and the articles that were published over 10 years with text availability as free full-text were included. A total of 119 articles were obtained, which were exported as Comma-Separated Values (CSV) files from the database and inserted into a Microsoft Excel (Redmond, USA) spreadsheet to remove identical articles and screen them. As a result, only a handful of articles were gathered, and the SCOPUS database was also explored. 

Several filters were employed in the SCOPUS database, such as the year of publication (from 2011 to 2021), subject area in which non-medical fields were excluded, document types that were limited to articles and reviews, and the selected language was English. A total of 196 articles were exported as CSV files and inserted into Excel (Redmond, USA) spreadsheets to remove identical articles and screen them.

Data filtration and analyzation

The identified studies were screened manually by two authors (SMG and KNM). Bibliometric analysis of reviews that were not based on case reports or case series were excluded. It is also important to note that any article on bibliometric analysis of a given journal that consisted of one or a few case reports, along with other types of articles, was ruled out.

**Figure 1:**
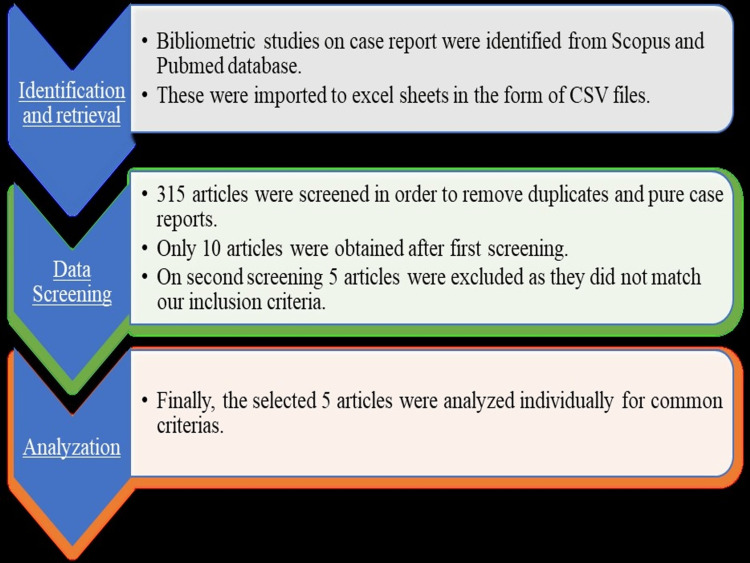
Flowchart depicting the process for conducting analyses on bibliometric studies of case reports. CSV: Comma separated values

**Figure 2:**
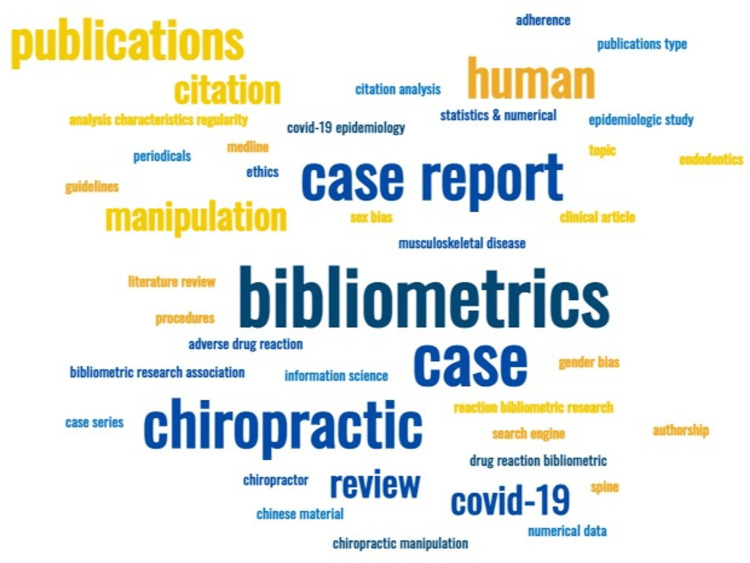
A visual representation of author keywords on bibliometric studies of case reports.

A total of 315 articles from both PubMed and SCOPUS databases were analyzed in order to remove duplicates and pure case reports, from which only 10 articles were selected. These were then imported to a new Excel (Redmond, USA) sheet for a final review, in which 5 out of 10 articles (Figure 1) were eliminated because some were systematic reviews, diachronic studies, etc. A visual representation of author keywords is shown in Figure 2.

Literature review

The selected articles were then examined individually for common criteria employed for bibliometrics. Year of publication, publication type, number of case reports analyzed per article, citation metrics along with impact factor (IF), subject of the article, author affiliation, and country were the parameters that were analyzed. 

**Figure 3:**
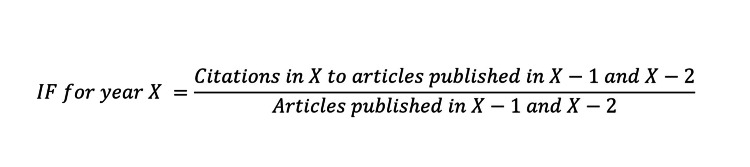
Formula to calculate Impact Factor IF: Impact Factor

The IF of an article is the total number of times an article in a particular journal has been cited in the previous two years. To calculate the article’s IF, the total citation sum was determined from the databases individually for each study, and the IFs were obtained using the following formula: The IF of a journal A in a particular year Y is computed following the formula which is mentioned in Figure 3.

The sum of “citable articles” in a given time period published in a specific journal is the denominator, while the numerator is the sum of citations in the present year to any article published in this specific journal during that given time period. This period was defined as two years by the International Statistical Institute (ISI) [8].

Other criteria were examined one by one manually and interpretations were drawn as shown in Table 1.

**Table 1:** Articles on Bibliometrics of case reports CMAJ: Canadian Medical Association Journal, USA: United States of America, UK: United Kingdom, UAE: United Arab Emirates.

Sr. no	Publication	Journal	Country	Author Affiliation	Year
1.	Sex and Gender Bias in Covid-19 Clinical Case Reports [3]	Frontiers in Global Women's Health	USA	Department of Medical Social Sciences, Feinberg School of Medicine, Northwestern University, Chicago, IL, United States.	2021
2.	Completeness of reporting for COVID-19 case reports, January to April 2020: A meta-epidemiologic study [4]	CMAJ Open	UK	Faculty of Health Sciences (Scaffidi), School of Medicine, Queen's University, Kingston	2021
3.	A bibliometric analysis of the top 100 most-cited case reports and case series in Endodontic journals [5]	International Endodontic Journal	UAE	Department of Preventive and Restorative Dentistry, College of Dental Medicine, University of Sharjah, Sharjah, UAE.	2021
4.	Chiropractic case reports: a review and bibliometric analysis [6]	Chiropractic & Manual Therapies	USA	Connor Integrative Health Network, Cleveland Medical Center, USA.	2021
5.	The characteristics and regularities of cardiac adverse drug reactions induced by Chinese materia medica: A bibliometric research and association rules analysis [7]	Journal of Ethnopharmacology	China	Department of Clinical Chinese Pharmacy, School of Chinese Materia Medica, Beijing University of Chinese Medicine, China	2020

Results

Here, the bibliometric studies were inspected based on common criteria, that is, year of publication, impact factor, citation, publication type, and the number of case reports per article from 2011-2021 to the period.

Publication by years

Five bibliometric articles on case reports were published between 2011 and 2021. As shown in Table 2, there were no publications published from 2011 to 2019 according to our inclusion criteria. Only one article was reported in 2020, and the number increased to four articles in 2021. 

**Table 2:** Publications by years on bibliometric studies of case reports.

Years	N	%
2019	-	-
2020	1	20
2021	4	80
Total	5	100

Type of publication

One of the five articles was a case report, and the rest four were review-type publications, among which one was a meta-analysis. 

Number of case reports per article 

**Figure 4:**
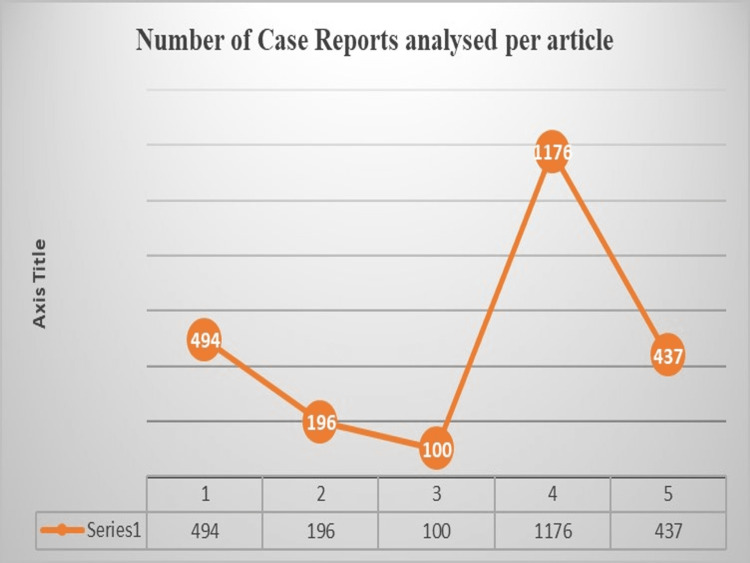
Number of case reports analyzed in each bibliometric article

A total of 2403 case reports were present in the selected criteria, out of which the article on Chiropractic and manual therapies had the highest sum of case reports (1176), and the lowest sum of case reports was found in the article from the International Endodontic Journals [5,6]. The number of case reports analyzed per article is graphically presented in Figure 4.

Articles according to themes/subjects

Of the five articles in our study, two (40%) were bibliometrics of case reports based on COVID-19, while three (60%) were based on other fields such as endodontic, chiropractic, and ethnopharmacology. The titles of four articles had phrases such as “case reports”, and two out of five had phrases such as “bibliometric analyses” as well as “case reports” case reports. 

Citation and Impact factor (IF)

**Figure 5:**
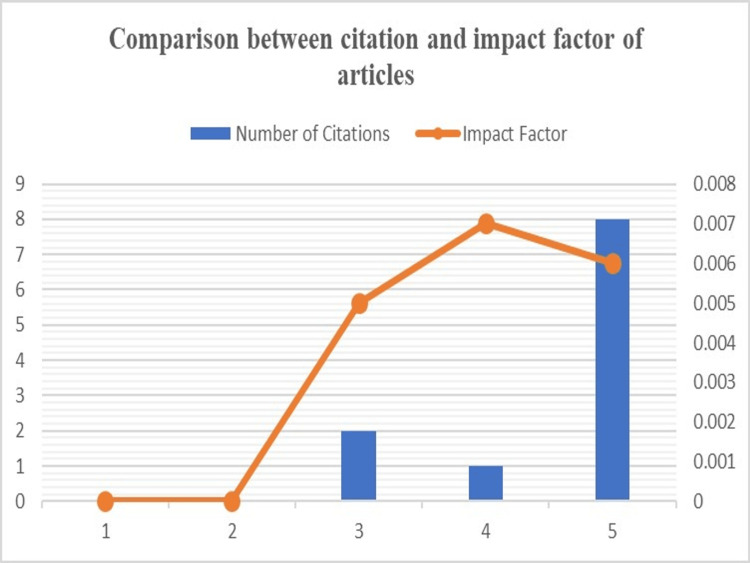
A comparison between the number of citations and the impact factor of published articles in their respective journals.

The total citations of different articles and their impact factor (IF) in the journal were found. Two out of five articles had no citations, and therefore the IF was 0 when calculated. The total citations ranged from 0-8, and the IF ranged between 0.00-0.007. A comparison between the number of citations and IF of published articles in their respective journals is graphically represented in Figure 5.

Discussion

The analysis helped us determine trends and their impact on other factors that are usually used to evaluate the quality of case reports bibliometrically. The progression of certain fields may be established through such parameters. Five articles were analyzed based on common parameters (year and type of publication, subject of articles, impact factor [IF], and citation). There has been a growth in the total number of article publications for the time period, with the maximum, i.e., four being in the year 2021. Of these four articles, two were COVID-19-associated studies. During the pandemic of the coronavirus disease, there was a high demand for immediate access to high-quality evidence provided by case reports, as these are the first published evidence for a disease and can detect novelties, offer insights into rare exposures, and generate hypotheses for future research [9,10]. 

One analysis had the highest number of case reports compared and was cited more often in chiropractic research. This implies that in the field of chiropractic medicine since more case reports have been published, there is a fair chance that one might find more information on rare diseases, unusual clinical symptoms, side effects of different treatments, and therapeutic effects of that field [6]. According to previous studies, a “classic” manuscript receives more than 400 citations, although the manuscript of minor specialties getting over and above 100 citations has also been regarded as a “classic” manuscript with finite authors and educational settings [11,12]. Thus the manuscripts which are “classic” are considered an acknowledgment that clinical and scientific communities have recognized them as a significant contribution to the specialty [11]. However, in this research, all the included articles received less than 10 citations and thus had a very low IF. This implies that these types of bibliometric articles neither receive enough acknowledgment nor have they made significant contributions to the clinical and scientific communities.

Our analysis had a few limitations. First, the screening of 319 articles was performed manually, which might be a reason for several errors that might have occurred in the screening and analysis of articles. Second, we only included case reports of all the fields receding within the medical sciences; hence, the number of articles finalized was very small because the data collected were not extensive. All articles that did not have free access, full text, and English as a language were excluded from the research; therefore, there is a fair chance that we might have not included some relevant articles due to our filtration criteria. Moreover, researchers could include other databases and different types of publications for future analysis. Additional keywords and parameters or the analysis of a specific domain along with sub-specialities pose the scope for future research, which will significantly contribute to the present research gap.

## Conclusions

This was the first study to carry out a bibliometric analysis of bibliometric studies of case reports in the field of medicine, providing an overview of the recent trends and criteria that are being adopted for conducting bibliometric implications of case reports. It has also been noted that there are not enough studies analyzing case reports that are the first line of evidence and are extremely important for the development of different branches within the medical field.

Therefore, this study contributes to this research gap to some extent and can be extended to various fields and specialties, which will allow the authors to investigate unique factors specific to their field of study. Analyzing the bibliometric articles of case reports in such a way will extensively provide some significant information about new trends, progress as well as the current direction in which that specific field is headed, thus having a major impact on the advancement of that particular field.
